# Supplementary material for: Towards a Neurophenomenological Understanding of Self-Disorder in Schizophrenia Spectrum Disorders: A Systematic Review and Synthesis of Anatomical, Physiological, and Neurocognitive Findings
Source: Brain Sci. 2023 May 23;13(6):845. doi: 10.3390/brainsci13060845 (PMC10296413; doi:10.3390/brainsci13060845)
Supplement: Supplementary file 1 [file brainsci-13-00845-s001.zip › brainsci-2406431-supplementary.pptx]

## Slide 1
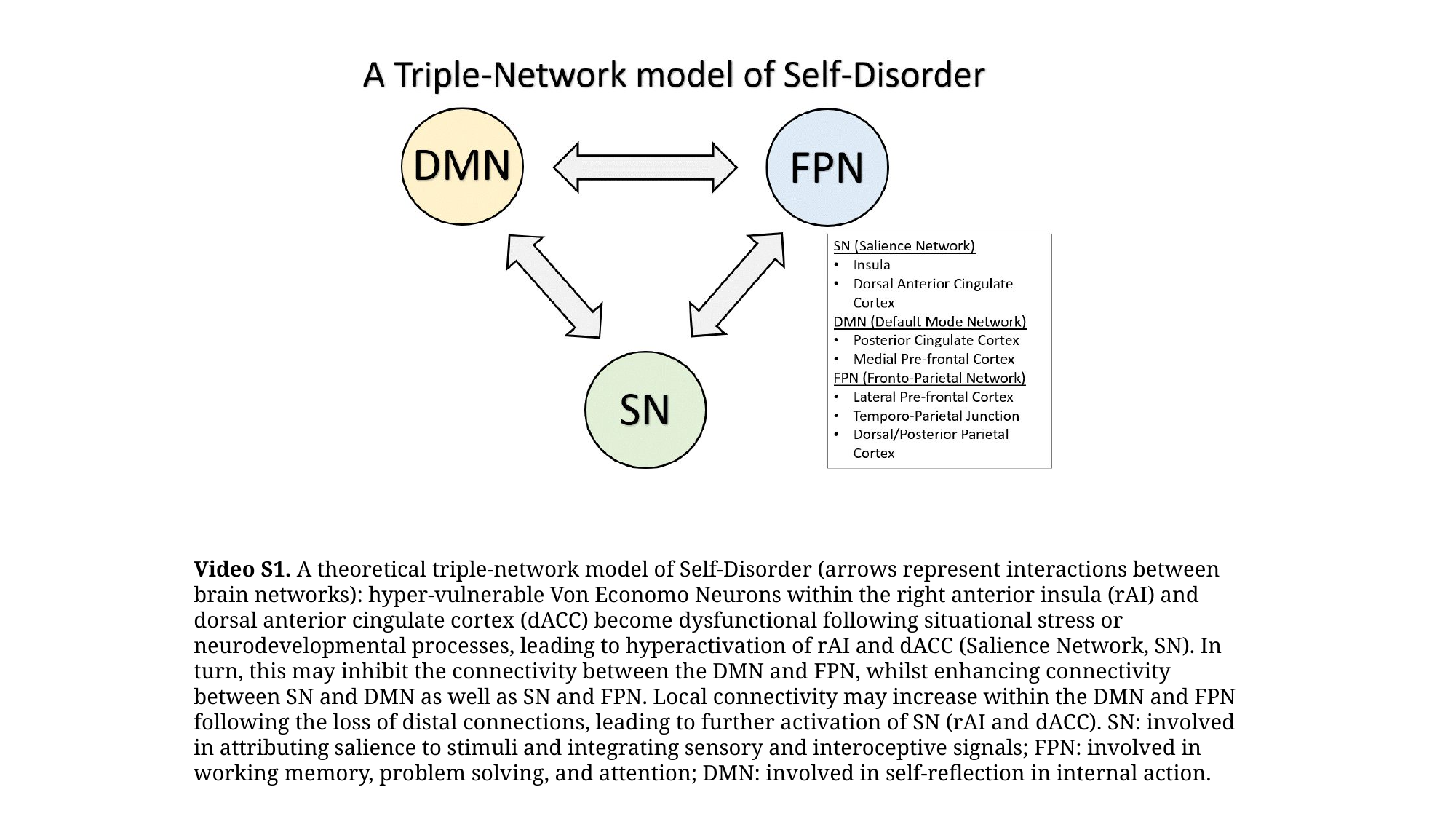

Video S1. A theoretical triple-network model of Self-Disorder (arrows represent interactions between brain networks): hyper-vulnerable Von Economo Neurons within the right anterior insula (rAI) and dorsal anterior cingulate cortex (dACC) become dysfunctional following situational stress or neurodevelopmental processes, leading to hyperactivation of rAI and dACC (Salience Network, SN). In turn, this may inhibit the connectivity between the DMN and FPN, whilst enhancing connectivity between SN and DMN as well as SN and FPN. Local connectivity may increase within the DMN and FPN following the loss of distal connections, leading to further activation of SN (rAI and dACC). SN: involved in attributing salience to stimuli and integrating sensory and interoceptive signals; FPN: involved in working memory, problem solving, and attention; DMN: involved in self-reflection in internal action.
